# Towards establishing human body-on-a-chip systems

**DOI:** 10.1186/s13287-022-03130-5

**Published:** 2022-08-20

**Authors:** Zhong Alan Li, Rocky S. Tuan

**Affiliations:** 1 Department of Biomedical Engineering, The Chinese University of Hong Kong, Shatin, Hong Kong SAR China; 2 Institute for Tissue Engineering and Regenerative Medicine, The Chinese University of Hong Kong, Shatin, Hong Kong SAR China

**Keywords:** Organ-on-a-chip, Body-on-a-chip, Patient-on-a-chip, Stem cell, Microfluidic device, Disease modelling, Drug testing, Precision medicine

## Abstract

Body-on-a-chip (BoC) platforms are established from multiple organs-on-chips (OoCs) to recapitulate the interactions between different tissues. Recently, Vunjak-Novakovic and colleagues reported the creation of a BoC system comprising four fluidically linked OoCs. Herein, the major innovations in their BoC system are discussed, followed by our future perspectives on enhancing the physiological relevance and scalability of BoCs for applications in studying disease mechanisms, testing potential therapeutics, and developing personalized medicine.

## Background

For the many diseases with incompletely understood pathogenic mechanisms, the development of safe and effective therapeutics requires the use of valid experimental and pre-clinical models prior to clinical trials on human patients. As the inadequacies of standard in vitro cell cultures are well-acknowledged, animal models are extensively used in biomedical research. However, because of inherent inter-species differences related to genetics, physiology, anatomy, metabolism, and other variations, a large number of drugs that show efficacy in animals do not work in humans, with some even displaying overt toxicity. The lack of valid and effective research models thus represents a primary hurdle to efficient drug discovery and development.

Organ-on-a-chip (OoC) systems have recently emerged as an alternative, highly promising model. As in vitro platforms engineered following tissue engineering and microfabrication principles, often with stem cells, OoCs are minimally but appropriately functional systems that recapitulate key phenotypical, physiological, and functional features of their native counterparts. With the development of OoCs that mimic different organs, human body-on-a-chip (BoC) systems have emerged as miniature replicas of, or at least part of, the human body, often consisting of platforms of multi-organs-on-chips of two or more coupled OoCs. They can phenocopy key aspects of the complex human physiology and recapitulate the interactions between an administered drug and various organs in the body. As tissue–tissue crosstalk plays critical roles in the initiation and progression of human diseases, human BoCs typically require the recirculation of a commonly shared medium that enables reciprocal communication between or among the organ components while allowing them to maintain their own identity. However, there are possible complications and technical challenges associated with the use of a common medium, in addition to the inherent different metabolic and nutritional requirements of different tissues, including altered tissue phenotypes, bubble generation and medium leakage at connections, and increased risk of contamination.

**Fig. 1:**
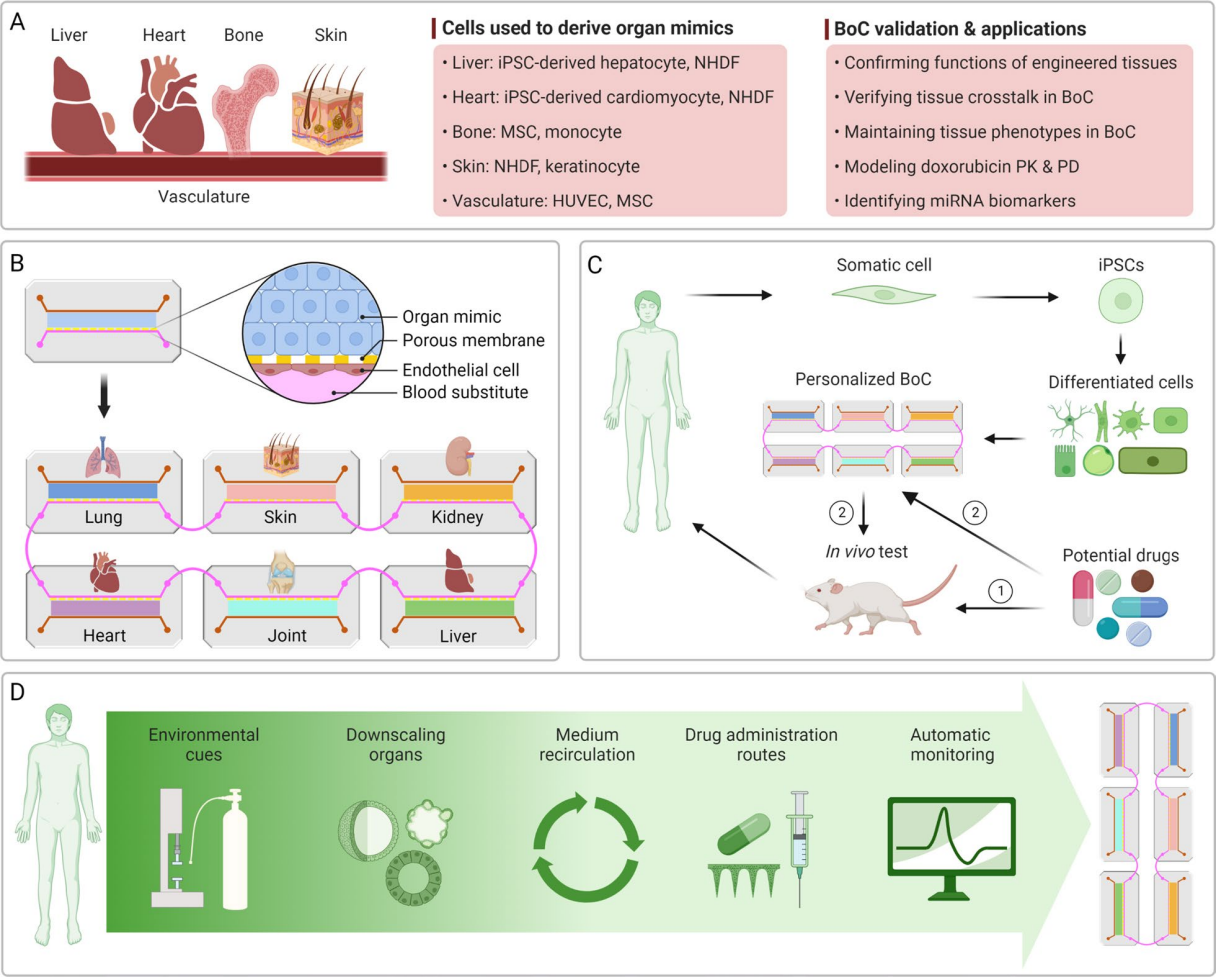
Representative BoC systems and future perspectives. (**A**) The BoC system created by Vunjak-Novakovic and colleagues: schematic of tissue components (left), the cell types utilized (middle), and validation and applications of the chip (right) [1]. NHDF: normal human dermal fibroblast; MSC: mesenchymal stromal cell; HUVEC: human umbilical venous endothelial cell. (**B**) A modular design approach to creating a representative six-organ BoC characterized by the sharing of a common “blood substitute”. The vascular channel is separated from the organ mimics by an endothelial barrier. (**C**) The application of iPSCs to generate patient-specific BoCs for personalized drug screening. By complementing the traditional drug development pipeline (conventional in vitro assays not shown) with personalized BoCs (from route ➀ route ➁), the ineffective and/or unsafe drugs for specific patients can “fail fast and fail early”, thus improving treatment outcomes at reduced costs. (**D**) Key considerations and technical challenges in establishing BoC systems

Recently, Vunjak-Novakovic and colleagues developed a BoC housing three-dimensional (3D), engineered heart, liver, bone, and skin that were fluidically connected via a commonly shared simulated vascular flow under the organ compartments (Fig. 1A) [1]. Most tissue components in the BoC were engineered with induced pluripotent stem cell (iPSC)-derived cells seeded in different biomaterials, such as fibrin (for heart and liver), decellularized bovine bone (for bone), and collagen type I (for skin). The body of the device, containing four tissue chambers, a perfusate flow channel, and a medium reservoir, was manufactured from polysulfone, a biocompatible thermoplastic with high stiffness, toughness, and transparency, using a computer numerical control milling machine. A standard glass microscope slide was utilized as the bottom to allow real-time imaging. A vasculoendothelial barrier was formed by seeding endothelial cells and mesenchymal stromal cells (MSCs) on a mesh insert and culturing them under an optimized hydrodynamic shear stress. The tissues and the vasculoendothelium were individually matured prior to integration. The vasculoendothelial barrier separated the organ mimics cultured in their respective optimized environment in the top chambers and the recirculating vascular flow at the bottom. Notably, within 24 h after inducing cryoinjury to the cardiac tissue, circulating monocytes could extravasate from the vascular flow to the injury site.

For validation purposes, Vunjak-Novakovic’s research group cultured the organ mimics in the BoC for 4 weeks and analysed them afterwards [1]. The tissue components maintained well their respective phenotypes over this period. Proteomic analysis results further confirmed the biological fidelity of the engineered tissues, which was comparable to that of adult tissues from humans. To demonstrate inter-organ communication, cardiomyocytes transfected with a green fluorescent protein (GFP)-labeled CD63 exosome reporter were used to engineer the heart tissues. After 2 weeks of culture, CD63 exosomes were identified in all tissues in the chip, including the vasculoendothelial barrier, suggesting that cardiomyocyte-secreted exosomes could pass through the selectively permeable vascular barrier and be internalized by other cell types. Therefore, the BoC could be utilized to characterize the transfer of cardiomyocyte secretome to different tissues in the body and investigate exosome-mediated communication between heart and other organs.

Finally, Vunjak-Novakovic’s group administered doxorubicin, a cancer drug with known cardiotoxicity in humans, to the BoC by introducing it into the vascular flow [1]. The measured concentration profiles of doxorubicin and doxorubicinol (metabolized in the liver from doxorubicin) matched the computational model of their pharmacokinetics [PK, including changes in absorption, distribution, metabolism, and excretion (ADME)]. The authors also demonstrated the capability of the BoC in modelling the pharmacodynamics (PD) of doxorubicin. It is noteworthy that clinically observed early miRNA biomarkers of cardiotoxicity were also identified in the doxorubicin-treated BoC.

A defining feature of the BoC system developed by Vunjak-Novakovic and colleagues is the presence of an endothelial barrier [1], representing the semi-permeable structure that plays vital roles in the ADME of drugs in vivo, thus recapitulating in vivo mass transport through the parenchymal tissue–endothelium interfaces (Fig. 1B). Similarly, in an earlier study [2], Ingber and colleagues established a BoC system consisting of an endothelium-lined vascular channel with separated parenchymal and vascular compartments that enabled sharing of a “blood substitute” by eight OoCs. Such an endothelium-based separation is critical to preserving the specificity of parenchymal compartments, especially when stem cell-derived lineages are employed, as the differentiated cells tend to display phenotypic instability.

To study PK/PD parameters with BoCs, the inclusion and interconnection of multiple organs are vital because drug toxicity has been frequently observed in organs other than the target sites. The importance of a liver organ compartment in BoCs partly arises from the fact that pro-drugs are commonly used, which are often metabolically activated by liver cells. A prominent advantage of BoCs lies in their ability to reflect organ responses to drugs that would be difficult, if not impossible, to observe in isolated cultures of cells and tissues. Further enhancement of the clinical relevance of the BoC reported by Vunjak-Novakovic and colleagues [1] may include the incorporation of other organ components, such as gut and kidney, that are known to significantly influence drug PK/PD in humans. It should be pointed out that the study by Vunjak-Novakovic’s group simulated only a single, albeit most common, route of drug administration, i.e. systemic administration, in their BoC [1]. Future applications of BoCs should also examine other routes of drug administration, such as topical application of drugs on the skin component and intraarticular injection of therapeutics for the treatment of degenerative joint disorders [3].

It remains challenging to recreate the complex, multifaceted integration of intricate structural and functional features of native organs in BoCs. A modular, “plug-and-play” design is highly desirable to allow convenient integration of different organ modules depending on the specific applications (Fig. 1B) [1–3]. A key consideration not fully addressed in previously developed BoC platforms is the need to accurately downscale the human body components. For example, the number and density of tissue-specific cells in organ mimics should reflect their relative abundance and distribution in the native organs, and the volumetric ratio of medium to organ/tissue-specific cells should ideally match the physiological ranges of tissue fluid-to-cell ratios in vivo. If incorrectly represented in the BoC, such factors can lead to erroneous drug PD/PK parameters and inaccurate estimates of effective and toxic dosages. Therefore, it is critical to validate results obtained from BoCs with data obtained from human patients.

The heterogeneity of many human diseases requires individualized investigation and treatment. To date, few BoC systems have been developed to address the unmet patient-specific medical needs. An essential step towards generating a personalized BoC (“patient-on-a-chip”) is using patient-specific cells (Fig. 1C) [4]. iPSCs reprogrammed from somatic cells derived from specific patients hold much promise in personalized disease modelling and drug development as they carry individual-specific genetic profiles. As phenotypically and functionally specialized tissues are key to recapitulating in vivo physiology and drug metabolism, effective and standardized protocols for the induction of iPSC differentiation and maturation into specific tissue lineages are absolutely necessary, currently a significant hurdle to wider applications of iPSCs. It should be pointed out that for many organs, the phenotypic characteristics of tissues depend on the presence of and the interactions with other tissues, e.g. the relationship between articular cartilage and subchondral bone [3], highlighting an additional, unique advantage of multi-tissue BoC platforms. In addition, blood and personal health data as well as information derived from patient-derived biopsies and tissue explants should be taken into consideration to strengthen the patient-specificity of BoC platforms. Furthermore, different applications will require the BoC platform to be adaptable and customizable, thus posing a significant challenge in establishing versatile BoC systems with validated tissue crosstalk as well as the ability to be adapted to suit application-dependent focus on specific features over others.

Finally, native tissues and organs are subjected to various biophysical stimuli, often superimposing, such as tensile, compressive, and shear stresses. These variables have yet to be applied to BoC tissue components. Moreover, most tissues in current BoCs are routinely subjected to considerably higher oxygen levels than their native counterparts, a limitation that needs to be overcome in future BoC designs (Fig. 1D). Simplified handling, automated monitoring, and online evaluation are all technically challenging, especially with a large number of organ modules, but are indispensable for BoCs intended for high-throughput drug discovery applications (Fig. 1D). Promisingly, the progress of development of such BoCs is being fuelled by technological advancements in robotics, sensors, and artificial intelligence [2]. For example, the use of liquid-handling robotics can enable the fluidic coupling of a large number of OoC modules, facilitating the establishment of complex BoC systems with high physiological relevance [5]. In addition, artificial intelligence can be employed to manage and analyse large data sets generated in high-throughput testing to extract useful information [6]. The information gained would be critical for deciphering the roles of different organ components and design features in varying applications, thus guiding the creation of versatile and adaptable BoCs.

## Conclusions

Engineered in vitro, human cell-derived BoCs are humanized and humane platforms that promise to open a new avenue for understanding tissue-, organ-, and ultimately body-level (patho)physiology, predicting the safety and efficacy of potential therapeutics, and developing personalized treatment regimens, while also helping to achieve the 3 R’s (Replacement, Reduction, and Refinement) in animal research [7]. Further developments of BoC technologies will allow unsafe and/or inefficacious compounds to “fail fast and fail early” in preclinical tests, thus increasing the success rate of drug discovery, reducing the cost of pharmaceutical R&D, and decreasing the length of time of bench-to-bedside translation.

## Data Availability

Not applicable.

## Abbreviations

3D: Three-dimensional
ADME: Absorption, distribution, metabolism, and excretion
BoC: Body-on-a-chip
GFP: Green fluorescent protein
iPSC: Induced pluripotent stem cell
MSC: Mesenchymal stromal cell
OoC: Organ-on-a-chip
PD: Pharmacodynamics
PK: Pharmacokinetics
